# Medical expenditure and its inequity for people with disabilities: Evidence from the CHARLS 2018 data

**DOI:** 10.3389/fpubh.2022.977150

**Published:** 2022-09-29

**Authors:** Shengxuan Jin, Ying Sun, Jun Tao, Lanlan Tian, Jiawei Lin, Dongfu Qian

**Affiliations:** ^1^Institute of Healthy Jiangsu Development, Nanjing Medical University, Nanjing, China; ^2^The Affiliated Hospital of Qingdao University, Qingdao, China; ^3^Center for Global Health, Nanjing Medical University, Nanjing, China

**Keywords:** disabilities, medical expenditure, inequality, two-part model, horizontal inequity index

## Abstract

**Introduction:**

Disabilities may raise heavy medical expenses and rich-poor inequalities. However, data is lacking for the Chinese older populations. This study aimed to measure socioeconomic inequalities in medical expenses amongst the Chinese adult 45 years or older, and explored the main determinants among different disability categories.

**Method:**

Data from the 2018 China Health and Retirement Longitudinal Study (CHARLS) were used. Disabilities were divided into five categories: physical disabilities, intellectual disability, vision problems, hearing problems, and multiple disabilities. The two-part model was employed to identify the factors that are associated with medical expenditures. Socioeconomic inequalities were measured by the concentration index (CI), and the horizontal inequity index (HI) which adjusts for health needs. Decomposition analysis was further applied to evaluate the contribution of each determinant.

**Results:**

Two thousand four hundred nineteen people were included in this study. The CIs and HIs of the expenditure were both positive. Amongst the varied types of medical expenses, the highest CIs were found for self-treatment expenses (0.0262). Amongst the five categories of disabilities, the group with vision problem disability reported the highest CIs and HIs for outpatient expenses (CI = 0.0843, HI = 0.0751), self-treatment expenses (CI = 0.0958, HI = 0.1119), and total expenses (CI = 0.0622, HI = 0.0541). The group of intellectual disability reported the highest CI and HI (CI = 0.0707, HI = 0.0625). The decomposition analysis showed that income (80.32%), education (25.14%) and living in the rural areas (13.96%) were the main determinants of medical expenses for HI amongst all types of disabilities.

**Conclusion:**

For five types of disabilities, our data shows that medical expenses concentrated in the richer groups in China. Income, education, and rural areas factors were the main contributors to the economic-related inequalities. Health policies to improve the affordability of medical care are needed to decrease inequity of medical expenditures for people with disabilities.

## Introduction

In the international community, Article 25 of the United Nations Convention on the Rights of Persons with Disabilities and the United Nations Declaration on Sustainable Development Goal 3 recognizes the right of people with disabilities to access health care and emphasizes quality and equity in health care (1, 2). The World Bank and the World Health Organization have also made recommendations to build a scientific foundation for people with disabilities in health-care areas (3). In the “Tutorial for Outline of the Healthy China 2030 Plan,” China also proposed safeguarding the health of people with disabilities, narrowing the disparities in primary health services and health levels between urban and rural areas, regions and populations, and promoting social health equity (4). Therefore, it has become the international community's consensus that it is necessary to pay attention to the health of and health-care quality and equity for people with disabilities. People with disabilities account for a large portion of the population in China, which is estimated to be include 85 million people, accounting for more than 6% of China's total population (5). This population is aging, rural, and poorly educated (6).

Compared to people without disabilities, people with disabilities are reported to be four times more likely to experience poor health (7). The World Report on Disability revealed that 51–53% of people with disabilities could not afford health care, whereas 32–33% of people without disabilities could not afford health care. Emphasizing affordability is an important reason why people with disabilities in low-income countries do not access needed health care (8). Obesity, diabetes, depression, cardiovascular diseases, and chronic diseases all have a two-way impact on disability, and as a result, people with disabilities face higher medical care needs, which increases their economic burden (9). Affordability is a critical component of equal access to health care, and it is becoming increasingly relevant in evaluating and improving health systems' performance (10, 11). Medical expenditure has been the second largest consumption expenditure in households with disabilities in China, and it has had an annual increasing trend (12). Therefore, health care for people with disabilities generally involves significant pressure, and important inequalities may arise in health services and expenditures.

In recent years, an increasing number of studies have been conducted on socioeconomic inequality in health services that have considered variables such as income level, education, occupational status, and living area. Empirical studies at this scope in China have focused primarily on the elderly and chronic disease population, and there are also studies on minority groups, such as children and migrant workers (13, 14). Guo et al. (15) explored socioeconomic inequalities among the chronic disease population, revealing significant differences in inequality related to urban living, higher education, economic status, and social participation. According to a study by Fan et al. (16), inpatient health service utilization was more concentrated among the low-economic respondents in the middle-aged and elderly population. The primary contributors to pro-low-economic inequity were economic status and lifestyle factors. For people with disabilities, health care disparities were also observed in factors that include race and ethnic group, low income or education level, rural residents, and the uninsured (17). Groups with different disability types experience different barriers in the environment and self-dysfunction, and this may translate into larger disparities for specific subpopulations (18). We assumed that their socioeconomic inequality has gradually been increasing recently, making health care inequality an important public health problem due to the large size of this population.

Medical expenditures can reflect health care utilization among people with disabilities and effectively reflect health inequality (19). A previous study investigated the inequalities in relative out-of-pocket (OOP) health expenditures and revealed a pro-poor inequality. Factors of age, education, gender, health insurance, and chronic illnesses increase the OOP health expenditure inequality (20). Xu et al. (21) demonstrated the equity differences in households' annual medical expenditures and OOP outpatient and OOP inpatient expenditures between urban and rural populations. This result demonstrated that equity in rural residents was worse than that of urban residents. Additionally, Najaf et al. (22) investigated inequality in Iranian households' dental care expenditures. Although fewer studies have documented inequalities in health services and expenditures for people with disabilities, the evidence suggests that socioeconomic inequalities exist for the disabled (23). However, this association is not well-understood among the Chinese population with disabilities and across different disability groups.

In the face of aging population development, the physical function of people with disabilities will decline, and the risk of chronic diseases or other diseases could be higher, which will further increase the expenditure on medical treatment and rehabilitation of people with disabilities as well as increase the expenditure inequality. In such a context, the aim of this study is to measure socioeconomic inequality in health services and expenditures and explore its primary determinant factors that contribute to inequity among different disability groups. Using data from the 2018 China Health and Retirement Longitudinal Study (CHARLS), we first estimated the medical expenses of people with disabilities and determined the influencing factors on medical costs related to socioeconomic factors. We then measured the socioeconomic inequality and explored its primary determinant factors. Targeted strategies and suggestions for protecting the basic rights and interests of people with disabilities in different disability types were provided to promote health equity and improve health conditions.

## Method

### Data source and study measures

This study used cross-sectional data from the CHARLS Wave 4 (2018). Counties, districts, and villages were selected by multistage and probability proportionate scale sampling method. A total of 150 districts and counties and 450 villages/communities were randomly selected within 30 provincial administrative units in China (24). The survey was conducted to analyze the problem of healthy aging in China and to promote interdisciplinary research on healthy aging. The baseline survey was conducted in 2011 and was followed up every 2 years. CHARLS obtained informed consent from all participants. The biomedical ethics committee of Beijing University approved the study, and the survey design and procedures can be viewed in detail in the original study documentation.

In the CHARLS database, the disability status was assessed with the question “Do you have one of the following [physical disabilities, brain damage/mental retardation, vision problem, hearing problem, speech impediment] disabilities?” Responses were categorized as “yes” or “no” (25). Participants with two or more types of disabilities were defined as multiple disabilities (26). A total of 2,492 people were selected for the six types of disability. After excluding 16 cases for missing values in age information and a speech impediment (*n* = 57), due to the low number of cases, a total of 2,419 cases were included in the analysis.

### Ethical approval

Ethical approval for collecting data on human subjects was granted by the institutional review board of Peking University (protocol code IRB00001052-11015). All participants provided informed consent prior to data collection.

### Variable selection

In this study, we used the basic characteristics, health status and medical service information of people with disabilities from the database of “Demographic Backgrounds, ” “Health Status and Functioning, ” “Health Care and Insurance, ” and “Income, Expenditures and Assets.”

**1. Dependent variable**

The medical expenses of people with disabilities are divided into outpatient expenses, inpatient expenses, and self-treatment expenses in the CHARLS database (27). Outpatient expenses refer to expenses incurred in outpatient visits or receiving on-site medical services in the past month; inpatient expenses refer to hospitalization expenses incurred in the past year; and self-treatment costs are defined as the amount spent in the past month on medication (not including prescriptions). Expenses include total amount, reimbursement portion, and out-of-pocket portion. In this study, the annual medical expenses are considered to be the sum of outpatient expenses, inpatient expenses, and self-treatment expenses directly related to the utilization of medical services for persons with disabilities, and the calculation method is the sum of outpatient expenses, self-treatment expenses multiplied by 12, and hospitalization expenses (27, 28).

In this study, we selected the three cost variables and the annual total medical expenditure variables as the index variables to analyze the medical expenditure of the group of people with disabilities. In addition, any expenditure in outpatient expenses, hospitalization expenses, and self-treatment expenses was recorded as the variable “had medical expenses.” We did not define any of the three as “no medical expenses.” Because the annual total medical expenses presented skewness distribution, it was processed by natural logarithm.

**2. Independent variable**

Socioeconomic variables for people with disabilities were divided into needs-based and non-needs variables to explore the factors affecting medical expenditure and health inequality.

***(1) Needs-based variables***

Needs-based variables are those related to the characteristics and health status of persons with disabilities that impact their medical or rehabilitation service needs (19). This study selected six main variables as measurement indicators: gender, age, self-rated health level, disability, chronic disease, and self-care ability (work and labor, and housework).

***(2) Non-needs variables***

Non-needs variables are those related to socioeconomic and external variables that influence the demand for medical or rehabilitation services for persons with disabilities (19). This study selected six main factors, including location, education, income level, family size, marital status, and medical insurance participation.

Because people with disabilities need assistance from their families, family factors are associated with the need for health services for persons with disabilities, so family size and marital status are included as variables. Regional and residential variables were introduced to reflect the development differences between eastern, central, and western China and between urban and rural areas, as well as the differences in medical costs and health equity among people with disabilities. Education is another essential factor in determining health status (29). A good educational background can promote better access to health resources and information, creating further health inequality. In addition, the participation of medical insurance can reduce the medical burden of some families and promote the release of part of the family's demand for medical services.

In this study, given that there are outliers in the composition of household income in the data, we replaced income status by economic level, which is reflected by per capita household consumption expenditure (30). Per capita household consumption expenditure refers to the household expenditure in the year before the survey, which is the average of the permanent household population evenly divided, excluding productive spending.

The respondents were divided into a “low expenditure group,” “medium-low expenditure group,” “medium expenditure group,” “medium-high expenditure group,” and “high expenditure group” according to the fifth level of per capita household consumption expenditure (2400.00¥, 4800.00¥, 8000.00¥, 14000, 00¥). Household consumption expenditure can directly reflect the family's economic status, making its objectivity better than the income index.

### Statistical methods

We conducted a descriptive analysis to describe the sample characteristics and to compute the means and standard deviation for continuous variables, frequency, and percentage of classification variables. The logarithm of total medical expense was expressed as the median [inter quartile range (IQR)]. Univariate analysis was performed for nominal variables using the non-parametric Mann-Whitney U-test or Kruskal-Wallis H-test, and for continuous variables using Pearson's correlation analysis.

The total annual medical expenditure was not normally distributed and showed more zero numbers. Zero medical expenses may be the actual medical expenses, or it may reflect the fact that some people did not seek medical treatment within 1 year. The samples with expenses were highly biased in the cost value. Duan (31) and Newhouse and Phelps (32) transformed and applied the two-part model proposed by Cragg (33) to solve the deviation of zero medical cost according to the characteristics of two-stage decision making in medical service utilization. Therefore, we used the Two-part model to analyze medical expenditure. First, a logit model was used to estimate the probability of medical expenditure. Then, a generalized linear model was used to estimate the amount of medical expense.

Concentration indices (CI) were used to measure the income-related inequality in health services and expenditures. A positive CI indicated that people of high economic status spend more on health care than people of low economic status, whereas a negative CI indicated the opposite (34, 35). Then the horizontal inequity indices (HIs) were presented following the indirect need-standardization process that helps to analyze the impact of SES on medical expenditures by controlling for need factors and non-need factors associated with health care utilization (36). Horizontal inequity studies the role of SES and considers the need for health utilization. This means the SES plays a role in the decision of utilizing medical expenditures for the same level of care needs due to health status (37). Positive values for the HIs mean that individuals with the same level of care needs have different medical expenditures due to their SES. Finally, the decomposition method of the CI was applied to measure the determinant factors contributing to inequity. The ordinary least squares (OLS) regression model was used to indirectly standardize the medical expenditure.

The unit of medical expenditures were expressed in CNY. When *p* < 0.05, the results were statistically significant. All analyses were performed using the statistical package STATA 14 and SPSS 23.

## Results

### Sample description

Table 1 shows the socioeconomic characteristics used in the analysis. A total of 2, 419 people were included with an average age of 68.12 and 2.563 in self-reported health. Regarding disability types, hearing problems had the largest representation (28.9%), followed by vision problems (19.4%) and multiple disabilities (18.8%). However, intelligence disabilities (16.7%) and physical disabilities (16.1%) were the least represented. Most of the respondents had primary school education (44.2%), followed by illiteracy (30.1%) and junior high school and above (25.8%). The income levels were roughly evenly distributed in the range of 18.7%−22.4%. Respondents suffering from chronic disease (61.7%) and living in rural areas (75.8%) reported being married (80.6%) and covered by medical insurance (96.2%).

**Table 1:** Sample characteristics and factors associated with the logarithm of total medical expense (*n* = 2,419).

**Variable/category**	**Logarithm of total medical expense (CNY)**		***P*-value**
	**Total (%)/Mean ±Standard**	**Median (IQR)**	
Gender
Male	1,162 (48.0)	7.97 (6.800, 9.090)	0.496^a^
Female	1,257 (52.0)	8.01 (7.090, 9.105)	
Age	68.12 ± 10.760	-	0.325^c^
Self-reported health^#^	2.563 ± 0.963	-	0.001^c^
Disability types^#^			0.001^b^
Physical disability	390 (16.1)	8.00 (7.090, 9.063)	
Intelligence disability	404 (16.7)	8.19 (7.090, 9.290)	
Vision problem	470 (19.4)	7.86 (6.870, 9.010)	
Hearing problem	700 (28.9)	7.78 (6.580, 8.958)	
Multiple disabilities	455 (18.9)	8.19 (7.090, 9.270)	
Chronic disease^#^			<0.001^a^
Yes	1,492 (61.7)	8.19 (7.090, 9.320)	
No	927 (38.3)	7.50 (6.400, 8.700)	
Able to work^#^			<0.001^a^
Yes	999 (41.3)	7.50 (6.400, 8.550)	
No	1,418 (58.7)	8.19 (7.090, 9.390)	
Able to do housework^#^
Yes	606 (34.5)	8.19 (7.090, 9.170)	<0.001^a^
No	1,153 (65.5)	7.68 (6.400, 8.590)	
Location			0.041^b^
Eastern region	749 (31.0)	7.63 (6.580, 8.700)	
Central region	1,002 (41.4)	7.78 (6.870, 9.040)	
Western region	668 (27.6)	7.78 (6.730, 8.715)	
Living area^#^			0.001^a^
Urban/town/rural-urban fringe	585 (24.2)	8.01 (7.090, 8.933)	
Rural areas	1,834 (75.8)	7.78 (6.580, 8.700)	
Education			0.132^b^
Junior high school and above	623 (25.8)	7.78 (6.818, 8.960)	
Primary school education	1,069 (44.1)	7.78 (6.580, 8.870)	
Illiteracy	727 (30.1)	7.78 (6.660, 8.700)	
Income level^#^			<0.001^b^
High	482 (19.9)	8.19 (7.090, 9.390)	
Medium high	465 (19.3)	7.86 (6.800, 9.015)	
Medium	453 (18.7)	7.78 (6.580, 8.810)	
Medium low	477 (19.7)	7.68 (6.580, 8.700)	
Low	542 (22.4)	7.75 (6.400, 8.700)	
Family size	2.30 ± 0.709	-	0.755^c^
Marital status			0.766^a^
Married	1,949 (80.6)	8.01 (7.090, 9.100)	
Other	470 (19.4)	7.91 (6.953, 9.170)	
Medical insurance			0.299^a^
Yes	2,327 (96.2)	8.01 (7.090, 9.100)	
No	92 (3.8)	7.78 (6.040, 9.210)	

^a^Mann–Whitney U test; ^b^Kruskal–Wallis H test; ^c^Pearson's correlation analysis; #: p < 0.05; and IQR, interquartile range.

### Regression results of medical expenses

Univariate analysis showed that there were statistically significant differences in the logarithm of total medical expenses among self-rated health, disability types, chronic disease, ability to work and do housework, living area, and income level variables (*P* < 0.05), as shown in Table 1.

Table 2 shows the expenditure probability and amount models to determine factors influencing medical expenditures. In Model A1, chronic diseases and self-rated health status significantly affected the choice of medical expenditure, and the odds ratio (OR) value was 0.735 and 2.023, respectively. After adding the non-needs variables (Model A2), the needs variables of age, self-reported health, disability types, and chronic disease and non-needs variables of the region, education, economic level, and marriage had significant effects (*P* < 0.05). The factors influencing the amount of medical expenditure were explored in Model II. Model B1 showed that self-reported health and chronic disease affected medical expenses (*P* < 0.01). After adding non-needs variables (Model B2), the ability to work and income level variables showed significant effects (*P* < 0.05).

**Table 2:** Regression results of the two-part model of medical expenditure for people with disabilities.

**Variable**		**Model I: Expense probability model**				**Model II: Expense amount model**					
		**Model A1**		**Model A2**		**Model B1**			**Model B2**		
		**OR**	**95% CI**	**OR**	**95% CI**	**Beta**	**Std. Error**	**VIF**	**Beta**	**Std. Error**	**VIF**
Needs-based variables	Gender: Male	1.090	(0.874, 1.360)	0.933	(0.727, 1.197)	−0.037	0.09	1.02	−0.120	0.098	1.25
	Age	1.008	(0.996, 1.020)	1.017***	(1.004, 1.030)	−0.004	0.005	1.01	0.001	0.005	1.17
	Self–reported health	0.735***	(0.645, 0.838)	0.728***	(0.637, 0.831)	−0.369***	0.057	1.20	−0.397***	0.056	1.23
	Disability types: Intelligence disability	1.422	(0.945, 2.139)	1.442	(0.950, 2.190)	0.283	0.169	1.95	0.306	0.169	1.98
	Vision problem	1.275	(0.885, 1.837)	1.251	(0.860, 1.820)	0.157	0.159	2.18	0.152	0.160	2.22
	Hearing problem	1.403*	(1.003, 1.962)	1.444*	(1.024, 2.035)	0.138	0.146	2.53	0.167	0.147	2.57
	Multiple disabilities	1.338	(0.891, 2.011)	1.387	(0.912, 2.107)	0.138	0.166	2.00	0.190	0.168	2.05
	Chronic disease	2.023***	(1.616, 2.532)	1.987***	(1.581, 2.497)	0.318***	0.097	1.05	0.262***	0.096	1.07
	Able to work	0.846	(0.627, 1.141)	0.833	(0.614, 1.130)	−0.229	0.12	1.82	−0.276*	0.119	1.84
	Able to do housework	0.855	(0.624, 1.172)	0.810	(0.589, 1.114)	−0.122	0.122	1.78	−0.102	0.121	1.80
Non-needs variables	Location: Central region			0.698**	(0.526, 0.926)				0.062	0.106	1.47
	Eastern region			0.722**	(0.532, 0.978)				−0.128	0.119	1.48
	Living area: Urban/town/rural-urban fringe			0.822	(0.611, 1.106)				−0.107	0.109	1.24
	Education: Primary school education			1.112	(0.824, 1.499)				0.088	0.120	1.86
	Junior high school and above			1.478**	(1.025, 2.133)				0.215	0.146	2.31
	Income level: Medium low			1.491*	(1.054, 2.110)				0.240	0.140	1.75
	Medium			1.601*	(1.112, 2.305)				0.283	0.154	1.75
	Medium high			1.524*	(1.061, 2.188)				0.444*	0.154	1.89
	High			1.103	(0.752, 1.617)				0.750***	0.161	2.04
	Family size			0.983	(0.844, 1.145)				0.118	0.071	1.10
	Marital status: Married			1.465*	(1.057, 2.032)				−0.025	0.130	1.16
	Medical insurance: Yes			1.365	(0.760, 2.452)				0.309	0.278	1.05
	Constant	1.942	(0.776, 4.863)	0.698	(0.182, 2.668)	8.799***	0.375	1.650	7.706***	0.561	1.65
	*R* ^2^	0.057		0.0724		0.091			0.126		
	*N*	1,616		1,616		1,128			1,128		

^***^p < 0.001, ^**^p < 0.01, and ^*^p < 0.05.

### Socioeconomic inequality in medical expenditures

Table 3 illustrates the CIs and HIs for outpatient, inpatient, self-treatment and total expenditures by different disabilities groups. The results suggested that different disability groups were analyzed following the same pattern. The CIs and HIs for medical expenditures were all positive, revealing that medical expenditures are more concentrated among socioeconomically advantaged households. The greatest CI in the total disabilities was for total medical expenditure (0.0268), followed by self-treatment expenses (0.0262), inpatient expenses (0.0206), and inpatient expenses (0.0122). Vision problem disabilities showed the highest CIs and HIs in outpatients (CI = 0.0843, HI = 0.0751), self-treatment (CI = 0.0958, HI = 0.1119), and total expenditures (CI = 0.0622, HI = 0.0541), whereas the highest CI in inpatient expenditures was the in intellectual disability group (CI = 0.0707, HI = 0.0625).

**Table 3:** Inequality and horizontal Inequity for medical expenditure with different disabilities groups.

**Variable**	**Physical disability** **(*****N*** = **390)**		**Intelligence disability** **(*****N*** = **404)**		**Vision problem** **(*****N*** = **470)**		**Hearing problem** **(*****N*** = **700)**		**Multiple disabilities** **(*****N*** = **455)**		**Total disabilities** **(*****N*** = **2,419)**	
	**CI**	**HI**	**CI**	**HI**	**CI**	**HI**	**CI**	**HI**	**CI**	**HI**	**CI**	**HI**
Outpatient	0.0489 (0.0194)**	0.0194 (0.0316)	0.0185 (0.0225)	0.0337 (0.0252)	0.0843 (0.0211)***	0.0751 (0.0189)***	0.0029 (0.0147)	−0.0090 (0.0154)	0.0464 (0.0283)	0.0729 (0.0274)***	0.0206 (0.0106)*	0.0206 (0.0102)**
Inpatient	0.0602 (0.0172)***	0.0184 (0.0280)	0.0707 (0.0354)**	0.0625 (0.0041)***	0.0149 (0.0170)	0.0117 (0.0124)	0.0062 (0.0090)	0.0187 (0.0097)*	0.0046 (0.0145)	0.0020 (0.0162)	0.0122 (0.0063)*	0.0083 (0.0060)
Self- treatment	0.0577 (0.0480)	0.0185 (0.0040)***	0.0358 (0.0106)***	0.0410 (0.01114)***	0.0958 (0.0172)***	0.1119 (0.0246)***	0.0266 (0.0087)***	0.0321 (0.0082)***	0.0108 (0.0105)	0.0161 (0.0098)	0.0262 (0.0047)***	0.0314 (0.0045)***
Total	0.0348 (0.0310)	0.0419 (0.0245)*	0.0056 (0.0236)	0.0178 (0.0178)	0.0622 (0.0238)***	0.0541 (0.0232)**	0.0344 (0.0181)*	0.0379 (0.0177)**	0.0015 (0.0278)	0.0014 (0.0255)	0.0268 (0.0107)**	0.0320 (0.0102)***

^***^p < 0.01, ^**^p < 0.05, and ^*^p < 0.10.

### Determinants of socioeconomic inequality in medical expenditures

The results of the decomposition analysis of the socioeconomic-related inequality in total medical expenditures for different disability groups are reported in Table 4. The results showed that income level was the most important factor in explaining pro-rich inequality in medical expenditures (80.32% for total disabilities), followed by education (25.14% for total disabilities) and rural areas (13.96% for total disabilities). This implied that an equal distribution of education and living in rural areas would cause decreases of 25.14% and 13.96%, respectively, in the socioeconomic-related inequality. In addition, self-rated health, chronic diseases, the ability to work, and age variables also showed different inequality influences on different types of disabled people.

**Table 4:** Decomposition of inequalities in total medical expenditure with different disabilities groups.

**Variable**	**Physical disability** **(*****N*** = **390)**		**Intelligence disability** **(*****N*** = **404)**		**Vision problem** **(*****N*** = **470)**		**Hearing problem** **(*****N*** = **700)**		**Multiple disabilities** **(*****N*** = **455)**		**Total disabilities** **(*****N*** = **2,419)**	
	**Concentration**	**Percentage**	**Concentration**	**Percentage**	**Concentration**	**Percentage**	**Concentration**	**Percentage**	**Concentration**	**Percentage**	**Concentration**	**Percentage**
	**index**	**contribution**	**index**	**contribution**	**index**	**contribution**	**index**	**contribution**	**index**	**contribution**	**index**	**contribution**
		**(%)**		**(%)**		**(%)**		**(%)**		**(%)**		**(%)**
**Needs-based variables**						
Male	−0.0077	1.16%	0.0314	−14.42%	0.0664	0.58%	0.0142	−1.55%	0.0124	−34.08%	0.0247	−0.91%
Age 60–74	−0.0137	−3.78%	−0.0883	−125.55%	−0.0449	−4.53%	−0.0119	−1.88%	−0.0153	27.10%	−0.0334	−8.35%
≥75	−0.0701	−0.75%	−0.0495	−7.49%	−0.1031	−3.19%	−0.0978	−6.89%	−0.1336	151.10%	−0.0927	−6.90%
Self-reported health: low	−0.0197	−2.82%	−0.0784	135.80%	−0.1453	14.91%	0.0121	2.05%	−0.0707	−200.44%	−0.0598	5.14%
General	0.0420	9.16%	0.0634	−160.76%	0.1060	−17.51%	−0.0072	−0.81%	0.0600	339.24%	0.0436	−11.66%
Good	−0.1111	8.82%	0.0145	−6.63%	−0.0402	2.46%	0.0561	−0.10%	0.3259	−14.51%	0.0147	−1.33%
Well-good	−0.0624	4.55%	−0.1326	50.17%	−0.2183	13.75%	0.0840	−5.36%	−0.0395	−68.54%	−0.0327	3.97%
Having chronic disease	−0.0209	−17.78%	0.0214	53.30%	0.0482	7.57%	0.0424	13.51%	0.0360	−257.24%	0.0276	14.31%
Able to work	0.0849	−19.50%	0.0811	−165.94%	0.0446	1.87%	0.0175	−3.24%	0.0251	58.07%	0.0438	−8.31%
Able to do housework	0.0317	0.23%	0.0457	26.07%	0.0132	−2.84%	0.0207	−5.82%	0.0358	11.04%	0.0275	−5.17%
**Non-needs variables**						
Income level	0.0699	112.22%	0.0670	−239.39%	0.0653	55.20%	0.0642	75.17%	0.0706	−388.94%	0.0667	80.32%
Location: Central region	0.0084	−1.45%	0.0259	4.85%	0.0255	−0.86%	0.0090	−1.36%	0.0118	41.53%	0.0137	−2.00%
Eastern region	0.1124	−12.60%	0.0001	−0.06%	−0.0057	0.35%	0.0679	−6.12%	0.0007	1.80%	0.0411	−5.33%
Rural areas	−0.1090	−1.09%	−0.0915	95.62%	−0.1165	17.22%	−0.1195	15.19%	−0.0945	90.13%	−0.1093	13.96%
Education: Primary school education	−0.0942	−4.92%	0.0404	34.79%	−0.0213	−0.46%	−0.0476	−4.76%	0.0438	−6.75%	−0.0208	−1.39%
Junior high school and above	0.2036	20.29%	0.1594	236.57%	0.3088	6.28%	0.2105	33.56%	0.2412	220.69%	0.2274	26.53%
Married	0.0185	9.02%	0.0405	171.95%	0.0335	−1.40%	0.0323	7.79%	0.0178	−167.96%	0.0291	9.71%
Medical insurance	0.0112	17.84%	0.0010	−0.20%	0.0122	−0.05%	0.0137	−3.81%	−0.0021	69.11%	0.0082	4.10%

## Discussion

People with disabilities are one of the most vulnerable groups, and meeting their needs for basic medical services has tested the delicacy of China's health system. Our study investigated the socioeconomic inequality in health services and expenditures among persons with disabilities of different disability types in China. Similar to the research on the inequality of other groups, there was also a pro-rich inequality among disabled people, and the inequality varied across disability types. The results provide evidence for health policymakers to promote health equity and optimize medical services for people with disabilities and across different disability groups. This study may be the first to measure the inequality in health services and expenditures within people with disabilities. In addition, national databases were utilized, and different types of disabilities were explored to develop more targeted results.

The concentration and horizontal inequity indices were positive, indicating a pro-rich inequality in medical expenditure, whereby people with high-economic status had more medical expenses than those with low-economic status. The income-related inequality results of people with disabilities differed from the inpatient service utilization of middle-aged and older adults (16), and the inequality was lower than in people with hypertension (38). This implied that access to health services for persons with disabilities requires a higher economic level, and some health policies for vulnerable groups may help alleviate disability inequities. Among the three types of medical expenses in the total disabilities, inpatient expenses have the least inequality, which was inconsistent with previous research results (39), whereas self-treatment expenses have the most inequality. This may be due to the fact that health insurance policies for people with disabilities pay more for reimbursement of the use of inpatient services. Moreover, the HI of self-treatment expenditures in the total disabilities was higher than in the CI, suggesting that economy-related inequality had a more significant impact on self-treatment services. People with disability-related and secondary or concurrent health problems may be more likely to require long-term treatment or have longer recovery times (40, 41). Given the limitation of economic income, this population may have a higher burden of self-treatment expenses, such as medical drug use and nursing care outside of hospitalization, aggravating this inequity in self-treatment costs.

This study found that visual disabilities faced the most serious inequality in the outpatient and self-treatment categories compared with other disabilities, while intellectual disabilities experienced the most inequality in inpatient inequality, emphasizing that the health care utilization of these two groups should be of great concern. The inequity for visual disabilities may have been due to the heavy cost burden of eye examinations and eyeglass prescriptions (42). Moreover, the income and insurance disparities also caused eye care access inequality (43). Visual impairment has become the leading cause of age-related disability (42). Therefore, attention should be paid to equity in preventing and examining vision problems in the elderly. High-quality community-based eye care should be delivered to improve the equity in access to eye services (44). In addition, public funding should cover routine eye examinations for those with visual disabilities over the age of 60 to reduce the burden of regular inspection in outpatient or self-treatment situations. As for intellectual disabilities, people face more obstacles in communication, biomedical complexities, and disability discrimination (45, 46), causing inequalities in their access to health care. In addition, the reluctance of family members to hospitalize those with intellectual disabilities and poor symptom recognition by caregivers may contribute to later hospital referrals and inpatient utilization inequalities for intellectual disability groups (47). Therefore, professional inpatient care and medical services for persons with intellectual disabilities should be strengthened, including mandatory education and training based on their behavior and biomedical complexities (46), to alleviate the unattended and undiagnosed problems.

The decomposition analysis indicated that the income variable contributed the greatest pro-rich inequality and significantly impacted the probability and amount of medical expenses. This result showed that the economic level was also the primary factor that caused unfairness in people with disabilities (48). This may be because people with disabilities who have better economic conditions have a strong affordability to pay for medical treatment and to cope with the cost burden. This improved financial situation may increase health-care costs because of the opportunity to choose better care. Therefore, we should strengthen the support for people with disabilities in health-care delivery and improve the medical assistance for the person who is poor, without self-care ability, and living alone. The education variable was the second most important factor positively affecting inequality. This outcome for people with disabilities could be explained by those with higher education levels having a higher level of health knowledge and paying more attention to their health input (49). The variable of living in rural areas has contributed to inequity for the poor. A relevant study showed that registered rural residents were related to a significant 13–40% increase in unmet health care needs for people with disabilities in China (50), and this may be attributed to the fact that people in economically disadvantaged rural areas require more health services. Therefore, the pro-poverty inequality in rural areas may also mean that individuals with more demand have received more health care.

Among the need variables, age and chronic diseases were primarily attributed to medical expenditure inequalities. The age variable was found to decrease the inequity in our study, and this agreed with the findings of the inpatient utilization inequalities (38). One explanation might be that aging leads physical function declines, aggravating their disability problems and making them require more health services. Moreover, an improvement in basic old-age insurance for the aging population also strengthens financial affordability and decreases expenditure inequalities (51). Chronic diseases have contributed to the inequitable outcome for the rich. Consistent with other studies (16, 52), chronic diseases are also associated with medical expenditures. Persons with disabilities likely have overlapping functional limitations or complex health conditions that present higher medical needs, thus leading to higher medical expenditure (53). Moreover, the coexistence of chronic diseases will increase the treatment of symptoms and families' financial burden (54). We recommend that attention should be paid to intensifying publicity regarding the health hazards of chronic disease and raising the health awareness of people with disabilities, and this would be conducive to alleviating the inequity of medical expenditures (48).

However, the regression results showed that medical insurance factors did not significantly affect medical expenses, revealing that the impact of medical insurance on medical expenses and inequality was limited. This result was different from the result of study on the accessibility of services for people with physical disabilities (55). This may have been caused by the low payment for disability protection and the limited reimbursement catalog for the disabled older population. A previous study pointed out that the rationality of different eligibility criteria for benefits within the social security system has been widely doubted and challenged (56), leading to the inequality of service utilization among middle-aged and older people. Thereby, China has implemented a long-term care insurance (LTCI) policy to alleviate the problems in the health care system caused by the aging population (57). This measure allows the government to develop new ways to consider social security for the disabled. Different types of long-term care based on the needs of disabled elderly should be integrated and promoted, and the LTCI should cover these services to afford coordinated and continuous health care for people with disabilities (58).

Interesting information was revealed from the comparison of the different types of disabled people. The marital status showed positive elasticity and a higher percentage contribution to the CI for intelligence and multiple disabilities, and this indicated that being married was significantly associated with expense consumption, especially in the intelligence and multiple disabilities groups. This may have been due to the physical function of the people with disabilities, making them more dependent on family care. A previous study showed that 80.73% of the disabled elderly rely on family members as their primary caregivers (58). It can be concluded that when the person has partner or family number support, health care utilization is higher. Therefore, we suggest increasing the diversity of social networks for intelligence and multiple disabilities groups to improve their ability to pay for medical care and withstand medical risks. Family caregivers can provide self-care training for the disabled to ensure their basic living and self-care ability and promote the rehabilitation of the mental, physical, and social functions of people with disabilities (59). Additionally, the different effects of socioeconomic factors in the different disability groups also indicated that precise, classified, and targeted efforts in health care should be made by considering these different demand-side health needs according to disability types (60, 61).

Our study has several limitations. First, this study was based on cross-sectional data. The results of this study reflected only the influencing factors of medical costs but did not explain the causal relationship among these factors. Second, recall bias may have existed as a result of the use of self-reported methods to collect medical history. Finally, the comparison between people with and without disabilities, trends in longitudinal data, and other aspects of health service utilization for people with disabilities should be further carried out in subsequent studies.

## Conclusion

This study explored socioeconomic inequality in health services and expenditures for people with disabilities and among different disability groups. The results indicated that the pro-rich inequities exist in medical expenditures utilization, and self-treatment expenses experience the most inequality. Attention should be paid to health care inequality for the visual and intellectual disability groups. The decomposition analysis revealed that income, education, and rural area of non-need variables and need variables of age and having chronic diseases were attributed to medical expenditure inequality. Targeted and effective evidence-based interventions should be implemented to enhance the affordability of medical expenditures and decrease inequality according to different disability types for people with disabilities.

## Data availability statement

The datasets presented in this study can be found in online repositories. The names of the repository/repositories and accession number(s) can be found in the article/supplementary material.

## Author contributions

SJ led the analysis of the data and wrote the first draft of the manuscript. YS, JT, and LT helped in data collection and cleaning. DQ, SJ, and JL contributed to the study design, interpretation of the data, and helped in the writing of the final draft of the manuscript. All authors contributed to the article and approved the submitted version.

## Funding

This work was funded by Major Program of National Fund of Philosophy and Social Science of China (Grant Number: 20AZD081) and the Excellent Innovation Team of the Philosophy and Social Sciences in the Universities and Colleges of Jiangsu Province -The Public Health Policy and Management Innovation Research Team.

## Conflict of interest

The authors declare that the research was conducted in the absence of any commercial or financial relationships that could be construed as a potential conflict of interest.

## Publisher's note

All claims expressed in this article are solely those of the authors and do not necessarily represent those of their affiliated organizations, or those of the publisher, the editors and the reviewers. Any product that may be evaluated in this article, or claim that may be made by its manufacturer, is not guaranteed or endorsed by the publisher.

## Abbreviations

CHARLS: China Health and Retirement Longitudinal Study
DAHE: Disability-Associated Health Care Expenditures
CI: Concentration Index
HI: Horizontal Inequity Index.
